# A Favorable Outcome of Grade 3 Follicular Lymphoma Treated With Surgery and Obinutuzumab Combined With Chemotherapy: A Case Report and Literature Review

**DOI:** 10.7759/cureus.23595

**Published:** 2022-03-29

**Authors:** Erinie Mekheal, Brooke E Kania, Sherif Roman, Nader Mekheal, Vinod Kumar, Leena Bondili, Michael Maroules

**Affiliations:** 1 Internal Medicine, St. Joseph’s Regional Medical Center, Paterson, USA; 2 Hematology and Oncology, St. Joseph’s Regional Medical Center, Paterson, USA; 3 Internal Medicine and Hematology, St. Joseph’s Regional Medical Center, Paterson, USA; 4 Hematology and Oncology, St. Joseph’s University Medical Center, Paterson, USA

**Keywords:** o-chop, r-chop, non-bulky dlbcl, grade 3, intestinal lymphoma, primary follicular lymphoma

## Abstract

Follicular lymphoma is the most common type of low-grade non-Hodgkin lymphoma and the second most common type of lymphoma. Primary extranodal follicular lymphoma is rare compared with nodular follicular lymphoma involving the gastrointestinal (GI) tract. There has been uncertainty regarding follicular lymphomas due to the heterogeneous presentation and severity in which they present. However, studies showed that patients diagnosed with primary gastrointestinal follicular lymphoma do not typically differ in their presentation from those diagnosed with nodular follicular lymphoma involving the GI tract. Furthermore, recent literature identifies that patients diagnosed with grade 3 follicular lymphoma tend to have similar genetic and molecular entities to those diagnosed with diffuse large B-cell lymphoma (DLBCL). Based on these results, current studies have shown that patients with grade 3 follicular lymphoma who are treated with anthracycline-based regimens have similar outcomes to those with diffuse large B-cell lymphoma. However, additional studies are warranted to demonstrate the benefit of managing grade 3 follicular lymphoma with more aggressive anthracycline/rituximab-based regimens. Here, we present a case of a 44-year-old male diagnosed with grade 3 follicular lymphoma involving the gastrointestinal tract, who demonstrated an excellent treatment response following therapy similar to the treatment of bulky diffuse large B-cell lymphoma despite a tumor burden size below 7.5 cm.

## Introduction

The World Health Organization (WHO) defined grade 3 follicular lymphoma as patients found to have greater than 15 centroblasts per high-power field (HPF), subsequently divided into grade 3a with identified centrocytes and grade 3b with centroblasts organized in a solid sheetlike pattern. Grade 3 follicular lymphoma can present in various manners, with varying clinical prognoses [1]. Thus, additional studies are warranted regarding prognostic features and decisions on when and how to treat this disease.

Multiple studies have shown that patients diagnosed with grade 3 follicular lymphoma tend to have similar genetic and molecular entities to those diagnosed with diffuse large B-cell lymphoma (DLBCL) [2-7]. This case aims to demonstrate the benefit of managing grade 3 follicular lymphoma with curative intent and elaborate on the importance of clinical presentation and histologic analyses as risk factors that warranted using more aggressive anthracycline/rituximab-based regimens for better long-term outcomes.

## Case presentation

This is a 44-year-old Hispanic male with a past medical history of chronic gastritis, *H. pylori* infection with confirmed eradication, and extensive smoking who presented to our facility complaining of sudden onset of left lower quadrant abdominal pain starting the morning of presentation. The pain was associated with nausea, non-bloody non-bilious vomiting, and non-bloody diarrhea. The patient added that he was experiencing multiple mild similar episodes, along with unintentional weight loss and a thin stool caliber for the last few years. He denied fever, chills, malaise, night sweats, hematemesis, hematochezia, or melanotic stools. On examination, the patient was afebrile. He had mild tenderness in the left upper and lower abdominal quadrants without any lymphadenopathy, hepatomegaly, or splenomegaly. Laboratory values showed a white blood cell count of 11.4 × 10^3^ µL with no band cells, hemoglobin of 15.4 g/dL, and lactate dehydrogenase (LDH) of 186 unit/L. The patient was found to be negative for HBV and HIV infection.

**Figure 1:**
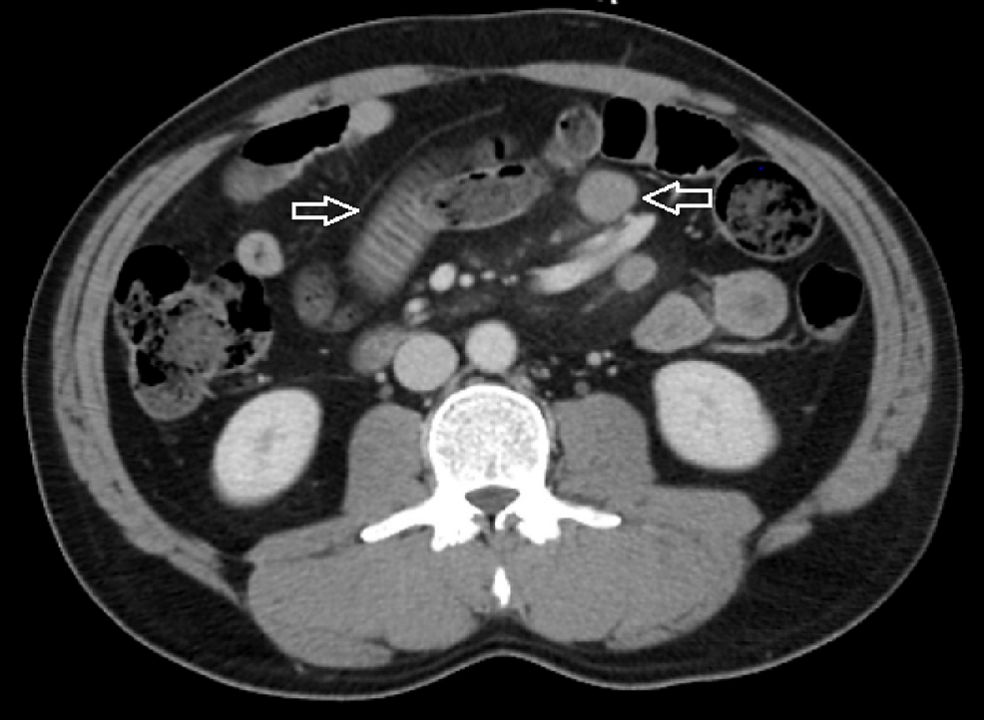
CT of the abdomen and pelvis showing multiple fecalized loops of small bowel without definitive transition point concerning for early/partial small bowel obstruction. There is mesenteric lymphadenopathy (measuring up to 1.9 cm) (right arrow) in conjunction with mural thickening of the small bowel along with slight aneurysmal dilatation of the lumen concerning for small bowel lymphoma (left arrow).

CT of the abdomen and pelvis with contrast showed partial small bowel obstruction (SBO), with mesenteric lymphadenopathy in conjunction with mural thickening of the small bowel concerning for small bowel lymphoma (Figure 1). Surgery was consulted, and the patient underwent diagnostic and therapeutic laparoscopy with two mesenteric lymph node biopsies, laparoscopic-assisted small bowel resection, and primary stapled small bowel anastomosis.

The gross description of the biopsies revealed one mesenteric lymph node measuring 2.7 × 2.6 × 1.4 cm, with the second measuring 3.4 × 2.2 × 1.8 cm. The midportion of the resected jejunal segment showed a slightly eroded hemorrhagic and congested mass-like surface, possibly representing the underlying mass that measures 5.0 × 4.5 × 1.8 cm. On sectioning, the mass was extended to the muscularis and involved the entire jejunum wall thickness and abutted the serosa but grossly not coming through the serosal surface. Upon examination of the serosal fat, three large grossly positive lymph nodes were identified, measuring 1.0 × 0.8 × 0.6 cm, 1.4 × 1.2 × 0.8 cm, and 1.8 × 1.6 × 1.2 cm.

**Figure 2:**
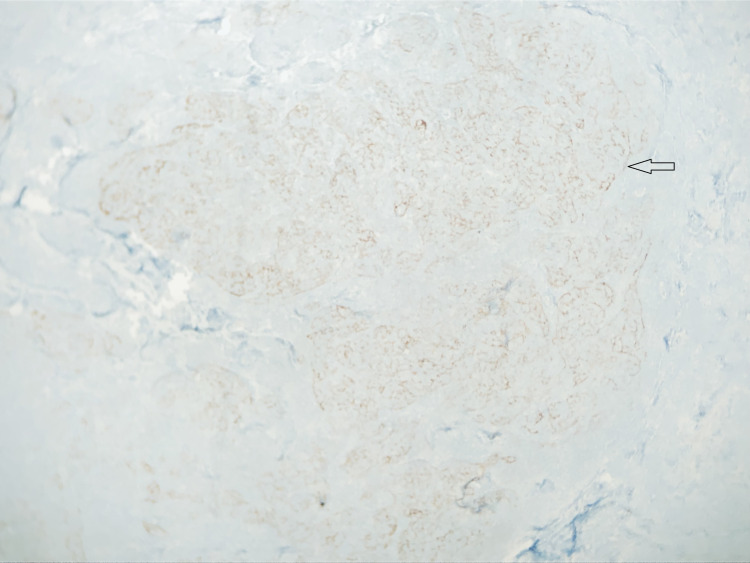
A stain with CD21 highlights the markedly expanded and disrupted residual follicular dendritic meshworks in the neoplastic follicles of the small bowel mass and the peri-intestinal lymph nodes (arrow).

The histopathological picture of the two mesenteric lymph nodes, the small bowel mass, and the peri-intestinal lymph nodes revealed atypical lymphoid follicles with immunostaining positive for CD10+, CD20, B-cell lymphoma 6 (BCL6), and BCL2 and negative for cyclin D1 and CD5. A stain with CD21 highlights the markedly expanded and disrupted residual follicular dendritic meshworks in the neoplastic follicles of the small bowel mass and the peri-intestinal lymph nodes (Figure 2). All these results were consistent with follicular lymphoma.

**Figure 3:**
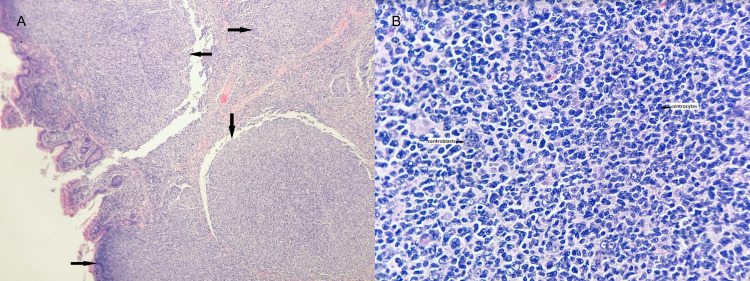
A: The atypical lymphoid nodules were variable in size, ranging from small to markedly enlarged, with attenuated mantle zones (arrows). B: Most lymphoid nodules’ lymphocytes were small to mildly enlarged centrocytes with cleaved nuclei consistent with follicular lymphoma. The centroblast counts in most of the neoplastic follicles were low (ranging from less than 5 to focally up to 15 HPF), indicating grade 1-2; however, a few small areas showed increased centroblasts (up to 20 HPF), indicating focally grade 3 according to the World Health Organization (WHO).

The pathological grade revealed follicular lymphoma mostly in situ (low grade) within the two mesenteric lymph nodes; however, the jejunal mass and peri-intestinal lymph nodes were consistent with extensive involvement by follicular lymphoma grade 1-2 and focally grade 3 (Figure 3A, 3B). Furthermore, phenotypic analysis by flow cytometry revealed a monoclonal population of CD19+, CD20+, CD10+, CD38+, and lambda positive B-cells (82% of the total cells), with negativity for CD5. The BCL2/IgH (t (14;18)) gene rearrangement was detected by fluorescence in situ hybridization (FISH), confirming the diagnosis of follicular lymphoma. While the location of the lymphoma raised the possibility of primary follicular lymphoma of the gastrointestinal (GI) tract, the extensive transmural involvement with large mass formation, focal high-grade features, and involvement of the mesenteric lymph nodes favored systemic follicular lymphoma involving the GI tract.

**Figure 4:**
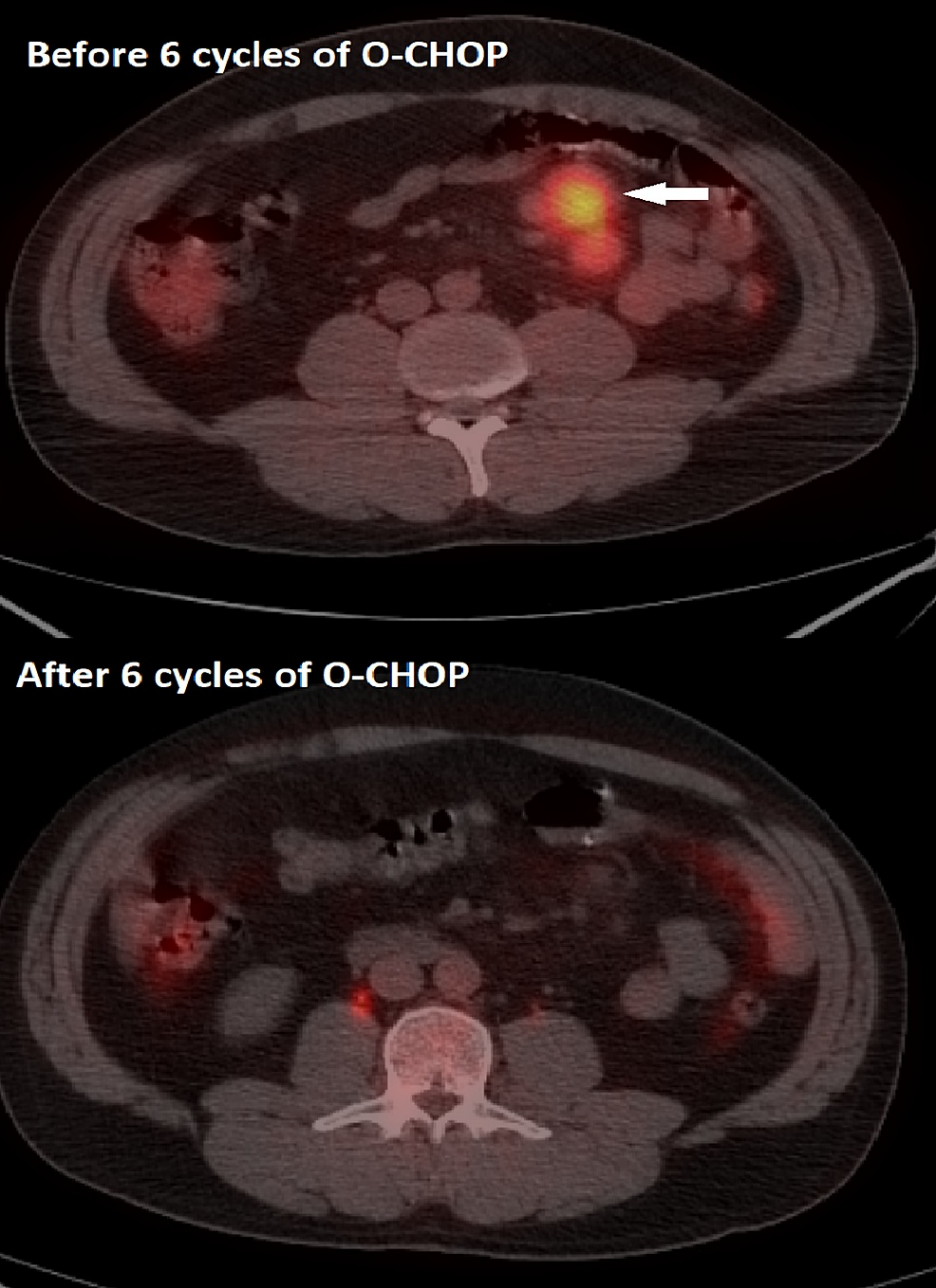
PET/CT scan before and after finishing six cycles of O-CHOP, showing resolution of the hypermetabolic mesenteric lymphadenopathy consistent with complete response to therapy.

Complete staging workup, including CT of the neck, chest, abdomen, and pelvis, bone marrow aspirate and biopsy, and positron emission tomography (PET) scan, were performed. PET/CT scan was significant for enlarged hypermetabolic lymph nodes in the root of the mesentery SUV 6.9. The was no other significant FDG uptake or adenopathy. According to the Ann Arbor staging system, the final staging of the tumor was stage II. The Follicular Lymphoma International Prognostic Index (FLIPI) was 0, indicating low risk and corresponding to a 10-year overall survival of approximately 70%. The Eastern Cooperative Oncology Group (ECOG) performance score was 0. The patient was started on rituximab-containing chemotherapy (R-CHOP) every 21 days with a plan to complete six cycles. However, as soon as the rituximab infusion was initiated, the patient developed an allergic reaction with skin redness and itching and ultimately did not receive CHOP that day. A decision was made to transition the regimen to obinutuzumab and CHOP (O-CHOP). The patient had finished six cycles of O-CHOP, and the repeat PET/CT scan demonstrated resolution of the hypermetabolic mesenteric lymphadenopathy, indicating a complete response to therapy (Figure 4).

## Discussion

In terms of the World Health Organization, follicular lymphoma encompasses a neoplastic disorder of follicular B-cells in a follicular pattern [1]. In the USA, among middle- to older-aged individuals, follicular lymphomas represent the most common type of indolent non-Hodgkin lymphomas and the second most common type of all lymphomas with a male-to-female ratio of 0.9:1.8 [2-7]. This type of malignancy can be found in splenic, hepatic, and bone marrow lymph nodes, and it is rare (<7%) for patients to present with primary extranodal follicular lymphoma [2-6]. In contrast, 66.7%-100% of patients present with nodular follicular lymphoma involving the gastrointestinal tract, especially in the jejunoileal segment, as in our patient’s case [8]. Further studies have shown that follicular lymphoma is usually diagnosed at an advanced stage III or IV according to the Ann Arbor staging system, with <10% of cases being diagnosed at an earlier stage I or II [9]. Our patient represented the minority of patients as a male, with nodular follicular lymphoma stage 2. However, he had a common presentation involving a common site of the gastrointestinal tract.

Gastrointestinal lymphomas represent 15%-20% of all malignancies occurring in the small intestine, with the ileum being the most common site (60%-65% of patients), followed by the duodenum (20%-25% of patients), and rarely the jejunum, as in our case [10]. For most patients, abdominal pain and intestinal obstructions have been the two most common presenting clinical manifestations [4]. Despite differences in prognosis, patients with both follicular lymphoma of either the jejunum or terminal ileum may present similarly and have similar endoscopic findings to patients with mantle cells; therefore, histologic analysis of the biopsied tissue is imperative for accurate diagnosis and management [4,11]. When comparing our patient, he had a common presentation of small bowel obstruction, and his molecular analysis was consistent with indolent follicular lymphoma. Further investigation of his cancer favored follicular lymphoma focally high grade 3, which made the management more challenging.

Although clinicians can utilize the FLIPI score to identify the prognostic predictors of follicular lymphoma, this score was created to help predict survival rates at the time of diagnosis and was not developed with the intention to use them for treatment selection or to predict survival after treatment has been initiated [8,12-14]. Despite the low FLIPI score in our patient, his index did not necessarily reflect survival or guide decisions for treatment options (Table 1).

**Table 1:** Our patient’s Follicular Lymphoma International Prognostic Index (FLIPI) score [8]

Follicular Lymphoma International Prognostic Index (FLIPI)	
Prognostic factors	Yes/no
Age > 60 years	No
More than four nodal sites	No
Lactate dehydrogenase (LDH) elevated	No
Hemoglobin < 120 g/L or 12 g/dL	No
Lymphoma stage III-IV	No
Final score	0 (low risk), 10-year overall survival is approximately 70%

Upon literature review, patients with high-grade follicular lymphoma can develop transformation to diffuse large B-cell lymphoma (DLBCL) with a variable transformation rate of 10%-60% [7]. Further studies have not demonstrated a significant difference in survival between patients with grade 3a and patients with grade 3b follicular lymphoma; the specific genetic manifestations of grade 3b follicular lymphoma may indicate a higher chance of transformation to DLBCL [7,15]. A prospective study of patients with grade 3 follicular lymphoma with lymph node positivity displayed no difference in event-free survival, overall survival, or presenting clinical characteristics between patients with histologic grade 3a, 3b, or C [16]. These findings warrant consideration to proceed with treatment for patients, especially those who are symptomatic. Our patient was symptomatic, as he presented with small bowel obstruction, leading to the decision to proceed with more aggressive management of his disease, although the current treatment for grade 3A follicular lymphoma is controversial [14]. In the post-rituximab era, patients with grade 3a or 3b follicular lymphoma showed a better prognosis regardless of the stage and/or patient’s age at the time of presentation [9]. Because of the differences in prognosis and therapeutic benefit of focal grade 3 lymphoma, it was decided to treat our patient with curative intent as DLBCL, ultimately preventing the aggressive transformation of this indolent lymphoma [14].

In continuing the challenge in treating our patient’s condition, studies reviewing the significance of extranodal maximum tumor diameter (EN-MTD) in the treatment of DLBCL have demonstrated that patients have a higher progression-free survival rate if their EN-MDT was <7.5 cm [17-19]. Our patient had promising prognostic factors because of his EN-MDT < 7.5 cm, but his less favorable clinical presentation of malignancy-related SBO eventually led the clinical team to treat him as if he had “bulky” DLBCL (defined as mass ≥ 7.5 cm) [14,17,18]. Ultimately, our patient received six cycles of R-CHOP (transitioned to O-CHOP due to tolerability), with a complete successful response to chemotherapy [14,18]. It is worth noting that the initial goal of developing a humanized type II monoclonal antibody (mAb) against CD20, also known as obinutuzumab or GA101, was the need for new treatments for patients with B-cell lymphomas, as well as alternatives for anti-CD20 drug hypersensitivity reactions (DHR) [20]. However, recent studies have shown that the combination of obinutuzumab and CHOP is more efficient than R-CHOP in providing a longer treatment-free period for relapsed lymphoma, all of which made the primary team confident of switching the patient to O-CHOP [20].

There is continued debate regarding consolidative radiotherapy following R-CHOP for this patient population [14,21]. A phase three, German, prospective, randomized trial of the Unfavorable Low-Risk Patients Treated with Densification of R-Chemo Regimens (UNFOLDER) evaluated patients with bulky (>7.5 cm) DLBCL who received R-CHOP with complete response regardless of whether they received radiation therapy (RT); however, patients who underwent RT were found to have improved overall survival [22]. In contrast, the British Columbia Cancer Agency has suggested for patients who have limited-stage DLBCL who had partial response following R-CHOP that RT would be beneficial, compared to those who achieved complete response [23]. The latest meta-analysis study on this topic suggests that more randomized trials are needed using contemporary R-CHOP and PET imaging to optimize therapy and minimize radiation-related toxicity [24]. Ultimately, as our patient showed a complete response to six cycles of R-CHOP, the decision was made to avoid radiation therapy as the risks outweigh the benefits of RT in his case.

## Conclusions

The purpose of this case study is to further comprehend the prognosis and treatment options for grade 3 follicular lymphoma. It is important to understand that follicular lymphoma is indolent in nature, but a certain subset of patients undergoes transformation to diffuse large B-cell lymphoma and ultimately has a worse prognosis. For these patients, it may be beneficial to treat for curative measures with a more aggressive treatment regimen similar to that of diffuse large B-cell lymphoma, such as anthracycline therapy with rituximab combination. It is important to note that each individual has different characteristics and risk factors, and therefore, it is important to make such decisions on an individual basis with the aid of histologic analyses. This holds true with our patient, who demonstrated excellent treatment response following therapy similar to the treatment of bulky diffuse large B-cell lymphoma despite a tumor burden size below 7.5 cm. In conclusion, additional studies are warranted in order to determine the appropriate treatment for such patients with grade 3 follicular lymphoma.
